# Healthiness as a Virtue: The Healthism of mHealth and the Challenges to Public Health

**DOI:** 10.1093/phe/phad019

**Published:** 2023-08-19

**Authors:** Michał Wieczorek, Leon Walter Sebastian Rossmaier

**Affiliations:** Dublin City University, Institute of Ethics, All Hallows Campus, Senior House, D09 N920, Dublin, Ireland; Section of Philosophy (BMS), University of Twente, Drinerlolaan 5, 7522 NB Enschede, The Netherlands

## Abstract

Mobile health (mHealth) technologies for self-monitoring health-relevant parameters such as heart frequency, sleeping patterns or exercise regimes aim at fostering healthy behavior change and increasing the individual users to promote and maintain their health. We argue that this aspect of mHealth supports healthism, the increasing shift from institutional responsibility for public health toward individual engagement in maintaining health as well as mitigating health risks. Moreover, this healthist paradigm leads to a shift from understanding health as the absence of illness to regarding health as the performance of certain rituals in order to project healthiness. By drawing from the analogy between healthiness and traditional virtues, we evaluate the promises made by proponents of mHealth technologies for self-monitoring. We argue that the implementation and use of mHealth risk entrenching existing inequalities and, more particularly, tend to exclude populations situated at the losing end of those inequalities from participating in the quasi-virtue of healthiness. Consequently, the implementation and use of mHealth technologies not only present challenges for social justice but also undermine their primary societal goal—to promote public health. Finally, we offer several suggestions on how to realize the potential benefit of mHealth.

## Introduction

Mobile health (mHealth) technologies are increasingly used by individuals and adopted in healthcare contexts. They allow users^1^ to self-monitor bodily functions and health-related behaviors outside regular care settings (Cvrkel, 2018; Davies, 2021). With the help of dedicated apps and devices, users are able to monitor their heart rate, blood oxygen level, blood pressure, mood or sleeping patterns without the support of medical professionals and independent from conventional clinical infrastructure. Patients can track their exercise routines, workouts or eating habits, while apps allow them to display, analyze and share the collected information within medical care settings, social networks or research contexts. The apps are easily accessible via the most popular app stores to anyone owning a portable digital device such as a smartphone or tablet and can be downloaded at comparatively low cost or free of charge depending on the provider’s business model. Moreover, dedicated devices promise greater accuracy of measurement than smartphones (e.g. Fitbit smart bands or Apple Watches) or enable the monitoring of more specialized metrics (e.g. continuous glucose monitors offer diabetics live reading of their blood sugar levels).

Such technologies can improve health at both the individual and public levels, with some claiming that their uptake would reduce the overall cost of providing health care, empower patients and increase access to health services. While we share the belief in the beneficial potential of mHealth, we argue that due to healthist attitudes that pervade and are promoted by such technologies, their ability to contribute to public health is limited. Admittedly, many authors have already discussed the moralization of health occurring within the healthist paradigm (see e.g. Crawford, 1980, 2006), especially in the context of technologies for self-monitoring (Lupton, 2013a, 2013b). However, we suggest that a discussion of healthiness through the lens of virtue can help us understand the limitations of mHealth as it is currently used and/or designed, and to overcome some of these issues in the interest of public health.

We develop our argument in the following manner. In the first section, we provide an understanding of public health and demonstrate the public health benefits of mHealth technologies by discussing three democratic^2^ promises surrounding mHealth. Second, we argue that healthist attitudes are embedded in and promoted by mHealth. Third, we outline some parallels between a healthist notion of healthiness and moral virtues as discussed in the (neo-) Aristotelian tradition to further develop this argument. Fourth, we claim that healthiness as a quasi-virtue presents an obstacle to the fulfillment of the democratic promises of mHealth. In the fifth step, we frame healthiness as a disposition achievable only by a select group of privileged individuals and demonstrate how structural injustices impact people’s ability to reap the benefits of mHealth. Finally, we discuss how to reflect the ideals of social justice and democracy in the design and deployment of mHealth and provide some examples of how to coordinate individual efforts in the pursuit of public health goals.

## Public Health Promises About Mobile Health Technologies for Self-Monitoring

Our understanding of public health is 2-fold. First, public health is concerned with promoting and protecting not only a population’s health in functional terms but encompasses a population’s well-being in a broader sense (Faden and Shebaya, 2015). According to this view, the population’s well-being includes physical and mental health-related aspects as well as a relational dimension that defines the requirements for a decent human life within society Powers and Faden (2019) and between individuals. Second, we view public health not solely as a top-down directed area of policy but as a participatory effort including the individual as a subject and active part in the concern for public health (Verweij and Dawson, 2007). Adopting this notion of public health will allow us to evaluate moral concerns raised by mHealth on an individual level in the context of its societal ramifications.

On the societal level, mHealth provides new opportunities for promoting, monitoring and researching public health outcomes (World Health Organization [WHO], 2011; European Commission, 2014). This potential has been widely acknowledged by public health experts in rich and poor countries alike (Barkman and Weinehall, 2017). Health insurance providers may offer mHealth technologies as loyalty program perks to their customers (SamenGezond, 2020) or even require their use as a condition for coverage (Barlyn, 2018). Public health organizations have issued calls to donate data from fitness apps to better predict new outbreaks of COVID-19 (Robert-Koch-Institut, 2021). Moreover, some employers, particularly in the USA, use mHealth technologies as part of workplace wellness schemes. In this context, employees’ uptake of self-monitoring tools is expected to reduce stress and the prevalence of occupational health risks, which could translate to an increase in productivity or a reduction of the employer’s insurance costs (Moore, 2017; Till, 2018).

Although the promotion of public health may not be the primary intention behind the development, deployment and use of mHealth apps for self-monitoring, these apps still follow a public health-relevant rationale. While engaging with an mHealth app is primarily an individualistic endeavor, mHealth apps bear the potential for facilitating the realization of public health goals, such as the prevention of health risks or big data-based public health monitoring. Moreover, public health organizations, such as the WHO, endorse the adoption of national plans to roll out mHealth interventions because it allows for engaging individuals in public health-relevant tasks (WHO, 2011). Therefore, the public health relevance of mHealth apps is rooted in their capacity to scale up individual health-oriented endeavors. Similarly, while mHealth mostly consists of consumer-grade tools offered by private technology companies and thus cannot be equated with health care as such, many healthcare systems and insurance companies closely integrate self-monitoring with conventional means of providing health or even use mHealth tools as their replacement (e.g. by relying on self-monitoring over clinical tests). Germany even went a step further and created a system for prescribing mHealth apps and devices, thus more strongly embedding mHealth within its healthcare systems (Sauermann *et al*., 2022). Consequently, while mHealth is not strictly health care, it extends beyond the sphere of consumer products as it is progressively being used in and treated as part of health care.

To outline our overarching concerns in this paper, we start by discussing the potential advantages provided by mHealth in the context of public health. We address three major democratic promises of self-monitoring for health—increased access to health care, empowerment and cost efficiency—but argue that the healthist paradigm within which mHealth functions is a major obstacle to their fulfillment. In our view, due to healthist attitudes, mHealth can have an undemocratic potential while also negatively affecting the well-being of users—particularly those who are already in a position of disadvantage.

The first promise of mHealth technologies for self-monitoring is to provide wider and easier access to health services, thus democratizing health care (Lucivero and Jongsma, 2017). This is of particular concern for persons bearing the consequences of digital, geographical or infrastructural inequalities. Wider and easier access to health services is supposedly guaranteed through the availability of apps downloaded via the internet. However, low prices, usability for individuals with low digital and health competency, as well as a functional internet connection are essential conditions to fulfill this promise.

The second promise states the potential of self-monitoring apps and devices for the patients’ empowerment. Empowerment is often understood as an improvement in self-knowledge and self-determination (Morley and Floridi, 2019), and as a greater independence from traditional care settings (Lucivero and Jongsma, 2017). The patients become more aware of their health and health risks and can use information from the app to mitigate symptoms, prevent illness and exercise more control in their relationship with health professionals. A certain degree of self-determination is one of the aspects of well-being and thus a necessary component for public health (Powers and Faden, 2006). However, considering that self-determination implies increased responsibility, it also connects to the previous promise and implies cost savings in the healthcare economy (Davies, 2021). Additionally, improved self-determination may affect other areas in life that contribute to the well-being of individuals that are of relevance for public health (Powers and Faden, 2006).

Finally, mHealth promises to increase cost efficiency within the healthcare sector and offers cheap solutions to facilitate preventative measures to avoid costs altogether (Lucivero and Jongsma, 2017). This aim is meant to be achieved through the introduction of design mechanisms aiming at supporting behavior change in the user. Thanks to self-monitoring, the user has permanent insight into their health and can adjust their behavior accordingly (Morley and Floridi, 2019). Consequently, patients face an increased responsibility to stay healthy, which is expected to lead to a reduction in spending within the healthcare systems without negative consequences for public health (Davies, 2021).

## Mobile Health Technologies for Self-Monitoring Within the Healthist Paradigm

In our view, the above-mentioned promises of mHealth technologies are offset by healthism and medicalization inherent in their design, marketing and deployment. As argued by Robert Crawford (1980, 2006), ever since the second half of the twentieth century, as well as the rise of neoliberalism, health and management of health-related risks have been increasingly delegated to individuals, shifting the responsibility away from various health and public health institutions. In the last 40 years, political and financial resources have been diverted from systemic means of addressing the problem of population health, and decision-makers have instead put effort into mobilizing individual citizens to assume a greater part of the burdens related to health and health-related risk management.

This individualization of health-related responsibility has been accompanied by a shift in our understanding of health in general. Within the healthist paradigm, health cannot be understood merely as an absence of illness, but as a performance of numerous rituals aimed at the projection of healthiness. Healthy individuals are expected to abstain from risky behaviors such as smoking and snacking on fatty foods, while simultaneously developing healthy habits including regular exercise, proper diet and increased monitoring of one’s body for the presence of unwanted symptoms (Swan, 2012; Lupton, 2013b).

Naturally, some degree of self-management by the patients has always been a part of the systemic provision of health care. However, as a result of healthist attitudes, unhealthy individuals are subjected to wide-reaching normative judgments. In the current era of individualized responsibility for health, ill health does not merely come from a patient’s bad luck, or is the result of a limited availability of resources within the healthcare system. Instead, unhealthiness is often framed as a personal failure—a direct result of one’s inability or lack of will to perform regular exercise, maintain a healthy diet and limit the influence of risk factors (Brown, 2018). In short, healthiness and health are, in the public imagination, closely tied to character and normative judgments. We will return to this point in the next section by drawing on parallels between healthiness and moral virtues.

mHealth technologies for self-monitoring have also been demonstrated to operate within the healthist paradigm (Lupton, 2013a, 2013b; Kleinpeter, 2017; Gabriels and Moerenhout, 2018). Patients who use mHealth apps are expected to put significant time and effort into managing their existing conditions, fostering healthy habits, and tracking and limiting their exposure to risk factors. This is particularly important when health-monitoring apps and devices are used within professional healthcare contexts or are tied to patients’ health insurance, as is increasingly common. In such situations, patients have little choice but to conform to the expectations placed upon them by healthcare and insurance providers, thus, perhaps unwittingly, assuming greater responsibility over their own health and engaging in the performance of health (sometimes not necessarily for health-related reasons, but merely to lower insurance premiums).

Moreover, users of mHealth technologies for self-monitoring often develop anxious and obsessive attitudes toward their health and are prone to medicalization, that is, treating even innocent and completely natural aspects of their daily life as indicative of underlying health issues or potentially unhealthy inclinations (Kreitmair and Cho, 2017; Lomborg *et al*., 2020). All data points are perceived as relevant for health monitoring, and users are trained by their devices to remain vigilant in order not to let undesirable (i.e. unhealthy) influences slip their attention.

## Healthism as a Virtue

As noted above, healthism links the assessment of an individual’s health (or healthiness) with an assessment of their behavior. In this section, we expand this claim to argue that healthism makes healthiness function in the public imagination similarly to (moral) virtues. And while we do not want to make the stronger claim that healthiness should be or is conceptualized precisely as a (moral) virtue, we argue that parallels between healthist attitudes toward health and the normative dimension of virtues are highly informative and can help us to analyze the promises and drawbacks of mHealth. Consequently, we discuss three key features of virtues as conceptualized in the (neo-)Aristotelian tradition and compare them with the notion of healthiness embedded in the healthist paradigm. This allows us to pinpoint factors that serve as an obstacle to the fulfillment of mHealth’s beneficial potential.

Ever since Aristotle’s (2004) influential discussion of virtues, the term can be understood as a stable disposition for specific kinds of action, or an excellent trait of character. Those willing to develop virtues should evaluate their character, identify existing inclinations, and engage in repeated action that would bring them closer to the desired state, often through imitation of those who are already virtuous. A virtuous individual is not necessarily defined by specific actions they undertake nor by their good outcomes (although they are bound to arise if said individual truly possesses the virtues) but by a set of habits pushing them in a certain direction.

In this way, virtues serve as an internal motivation for a certain kind of behavior (‘I act courageously because of my courage’), but they also possess an interpersonal normative dimension (Annas, 2015). Others are capable of evaluating the character of a given individual and forming expectations concerning their actions, praising or blaming them depending on the perceived presence or lack of a specific virtue (e.g. ‘Mark is a coward, we should not expect him to do the right thing’).

Finally, in the virtue ethical tradition, virtues are a key component of the good life—it is not possible to flourish as an individual and in a community without possessing the virtues. This is particularly well expressed by MacIntyre (2007), who argued that individuals (and their roles in communities) are defined not just by their excellence in morally salient practices. To make sense of their dispositions, as well as their relationship to some notion of the good life and the wider community, people share and construct narratives that express how certain aspects of their lives contributed (or not) to their flourishing. In this sense, the stories we tell about ourselves help us understand and communicate our moral experiences. Morally relevant factors will usually find their way into descriptions of important moments in our lives and into the narratives about our lives understood as unified wholes. When answering questions like ‘who are you?’, ‘what do you do?’ or ‘what you have been up to?’, we often give expression to the moral values that play a central role for us. Even simple responses to these questions, such as ‘I am a medical doctor’, ‘I teach philosophy’, ‘I haven’t done much recently, because I have been really tired’, can hint at the speaker’s ideas about the good life and their relationship with these ideas. Consequently, the analysis of values and sentiments expressed in narratives can help us make sense of moral beliefs and attitudes expressed by an individual or present in the society as a whole (see also Reijers and Coeckelbergh, 2020, for an in-depth analysis of the role of narratives in virtue ethics).

Healthiness, as we understand it and as framed within the healthist paradigm, bears close resemblances to a virtue, as characterized above. First, healthiness is arguably more of a matter of character rather than of particular actions or specific health-related outcomes. Healthy individuals possess a set of habits, but these habits do not necessarily translate into the absence of illness. While a significant proportion of people who maintain a healthy diet, regularly exercise, and abstain from drinking or smoking are more likely to possess good health, some outliers show that it is the character of the individual and not good health that defines healthiness within the public imagination. It is not uncommon to hear stories about somebody’s friends and family members who despite living a healthy life (i.e. demonstrably engaging in the practices of healthiness) eventually succumbed to a serious illness. These stories are most often framed as a matter of bad luck that occurred to an otherwise healthy person. For most, becoming ill is not incompatible with remaining healthy (in the healthist sense). Arguably, those who happen to become ill are often even more encouraged to engage in healthy behavior. Perhaps, in parallel to the belief that those possessing virtuous character are more likely to bring good outcomes through their actions, it is commonly accepted that the individuals exhibiting the virtue of healthiness are more likely to enjoy healthy outcomes, even despite the circumstances. Conversely, in the case of those who are considered unhealthy, regardless of their actual health, illness is more likely to be seen as a sign of their inferior habits. In such situations, it is the character of the person, and not bad luck that is blamed for the deterioration of health (similarly, a coward is more likely to be blamed for a failed courageous action, whereas a brave individual’s failure would be attributed to bad luck or strong competition).

Second, the normative aspects of healthiness are similar to the normative aspects of virtues. While healthiness is perceived as a character trait internally valuable to specific individuals, others are also capable (and it is conventional) to form normative judgments regarding one’s healthiness. Demonstrable healthy habits are commonly a reason for praise, just as their lack is met with admonishment and social disapproval. As argued above, healthiness is seen by others as a predictor of specific health outcomes but often also serves as a basis for a positive evaluation of an individual’s overall moral character. Conversely, unhealthiness is associated with moral failings in other areas, such as laziness, unreliability, irresponsibility or lack of temperance.

Third, healthiness is seen as a necessary component of the good life. In the popular imagination, narratives about human flourishing and happiness almost always contain references to health, but healthiness is also commonly framed as a characteristic of a life worth living. Contemporary media are saturated with aspirational stories about healthy individuals and members of the public are encouraged to pursue healthiness as a good in itself, but also as a set of habits contributing to their overall happiness (of course, this encouragement may also be seen as driving consumption of health-related goods and services and forms a significant part of mHealth’s marketing, see e.g. Apple Newsroom, 2021). The practices of healthiness (and excellence in them) occupy a central part in contemporary narratives about the good life. At the same time, skeptics may argue that what matters today, is not necessarily healthiness (or even health), but a possibility of narratively framing one’s behavior as healthy—the ability to portray oneself (to oneself and others) as a healthy, and thus praiseworthy, individual.

## Healthism as a Threat to the Promises of Mobile Health Technologies for Self-Monitoring

In addition to the characteristics discussed in the previous section, the healthist notion of healthiness resembles a moral virtue in yet another crucial sense. As we demonstrate below, not everyone is capable of adequately engaging in the pursuit of healthiness as the ability to develop healthy dispositions is co-determined by individual and societal/structural factors.

In the virtue ethical tradition, moral agents differ in their ability to develop and exercise their virtues. The opportunity to flourish is limited by the circumstances and individuals might not be able to become virtuous if they are constrained by factors such as money, care responsibilities, health or lack of political agency (Aristotle, 2004: 198; Annas, 2011: 146–168). After all, Aristotle’s ethics reflects the point of view of moral agents belonging to the aristocracy (see Honneth, 1998) and accepts that the members of the general public can only aspire to, but are unlikely to attain, the level of virtuousness and happiness available to the select few. As Aristotle himself notes (2004: 1178a), a slave is unlikely to be happy in the proper sense.

Similarly, healthiness (both in terms of actual performance of healthiness and the construction of narratives about it) appears to be an aristocratic pursuit, radically at odds with the democratic promises of mHealth. In our view, mHealth reflects the needs and perspectives of the most privileged members of the society. Due to this elitist outlook, and the virtue-like idea of healthiness, we argue that such technologies might function in an anti-democratic manner, even despite the best intentions of the people responsible for their design and implementation. We see several reasons why that may happen.

First, the successful use of mHealth technologies for self-monitoring requires a degree of health and digital literacy (as well as general hermeneutic capabilities or practical wisdom). These abilities are not equally distributed, and especially in the case of health literacy, they are more likely to be possessed by those who are already healthy (in the healthist sense).^3^ Patients failing to exhibit healthiness and engage with their apps and devices to the desired degree (often for structural reasons, such as limited access to the internet, or lower education level) require more help from healthcare professionals, similar to how those that have not yet acquired the virtues can benefit from education (Gabriels and Moerenhout, 2018; Klugman *et al*., 2018). However, as a large portion of health management is relegated to mHealth and to the patients themselves, these patients would lose out on vital contact with healthcare professionals, who would ordinarily offer health-related advice and guidance. In this sense, healthcare systems’ overreliance on mHealth would negatively impact the less fortunate members of the public and widen health inequalities even more.

Second, the individual benefits of mHealth technologies for self-monitoring, often discussed under the notion of empowerment, are similarly undermined by the undemocratic, virtue-like view of healthiness dominating the healthist paradigm. As already noted, users are expected to possess a degree of self-determination and readiness to assume responsibility over their health. However, just like virtues and the propensity to develop virtues, these characteristics are not equally distributed among the members of the public. mHealth technologies have already been criticized for contributing to ‘rich get richer’ effects as those with more resources, time and practical wisdom are more likely to reap their benefits (Gabriels and Moerenhout, 2018). However, we argue that this effect is even more pernicious in the context of institutionalized use of mHealth in healthcare systems. If the quasi-virtue of healthiness is assumed as the standard set of dispositions possessed by the users, systemic solutions are going to be designed in ways that account for the perceived distribution of healthy habits within the population. It is likely that mHealth-dependent healthcare systems will refer more to potential empowerment benefits rather than to professional interventions in individual health. Consequently, while it is certainly true that the rich may get richer while using mHealth technologies for self-monitoring, the poor might also get poorer, and not just in relative terms as they are significantly more likely to face unfavorable trade-offs connected to potential positive health developments (Rossmaier, 2022). For example, users coming from less-privileged backgrounds and more dependent on traditional care, might end up losing access and resources previously available to them if mHealth products are implemented as a replacement for conventional care services. Thus, they might be doubly disadvantaged as a result of the allocation of medical resources. First, because they cannot sufficiently access mHealth products, and second because other conventional options may fall prey to digitization processes and cease to be widely available. Unfortunately, this is bound to affect poorer people’s ability to engage in health-related practices and thus would widen the gap between them and the users who find themselves closer to the healthist ideal.

Third, as we already noted, these technologies aim at changing their users’ behavior to reduce the overall health costs, thus increasing the efficiency of healthcare systems. By promoting healthy habits, mHealth would reduce the number of expensive medical examinations, contact hours with medical professionals and costly treatments. However, these costs do not disappear. Instead, they are offset to individuals, who, as already noted, are increasingly expected to invest time, money and other resources to assume responsibility over the management and performance of their own health. However, not all users are equally able to engage in the practices of healthiness and pre-existing inequalities of resources might result in a proportionally greater burden being placed on the less-privileged members of the society (Owens and Cribb, 2019). While the promise of reduced healthcare costs is instinctively appealing, it assumes healthiness (in the virtue-like sense) to be the baseline set of habits and dispositions attainable to all. Many users struggle, however, to meet the standards applicable only to a small group of people and incur high financial, emotional and temporal costs to close the gap, often with mixed results. In addition, as resources are shifted away from systemic healthcare interventions, the less-privileged groups encounter complications when trying to access vital care (Kleinpeter, 2017). At the same time, they face numerous normative judgments related to either their perceived unhealthiness, or the amount of work they need to do in order to maintain the quasi-virtue (since, once acquired, virtues are seen to function automatically and without additional effort).

Additionally, although the adoption of smartphones is similar across income and education levels, age and gender (Pew Research Center, 2021), the use of mHealth apps and fitness trackers deviates from this trend. US-sourced data demonstrates that while in 2022 43% of high-income households have adopted health apps, only 27% of low-income households are mHealth users (Statista, 2022). Similarly, a survey of 4272 US adults suggests that only 31% of households earning 75,000$ per year or more use wearables and fitness trackers, while only 12% of households earning 30,000$ per year or less own such devices (Vogels, 2020). Using wearables is, however, often necessary to collect more accurate data and unlock all the apps’ features. Aside from differences in the adoption of mHealth apps, income inequalities also determine the modalities of use independent of the level of the users’ digital literacy. Low-income households are less capable of spending financial resources on premium subscriptions that would unlock features allowing more efficient and less invasive use of the products. Thus, they are at risk of being financially excluded from the more effective and efficient mHealth options, which limits their capacity to live up to healthist expectations.

On a related note, the above arguments assume that mHealth technologies work as advertised, which is not always the case. According to Piwek *et al*. (2016), there is simply not enough evidence to conclude that mHealth actually leads to the expected benefits. Empirical literature provides numerous examples of devices that fail to capture reliable metrics even when it comes to simple parameters such as daily step counts (Crawford *et al*., 2015) or heart rate (Lomborg *et al.*, 2020), and this is even more problematic when mHealth devices are used to monitor more complex factors such as mental health (Xie *et al*., 2022). Moreover, the authors note that many devices tend to break down, interrupting the continuity of monitoring or erasing the users’ data in the process (Klugman *et al*., 2018; Kristensen *et al*., 2021). Such shortcomings further problematize the concerns we raise in the context of wealth inequalities and their impacts on the beneficence of mHealth. It may be the case that only well-situated users will be able to afford mHealth technologies that offer a level of accuracy and reliability that would guarantee a positive impact on their health. In this sense, the technological shortcomings of mHealth tools would make the attainment of health (in the healthist sense) possible only to a select few, if at all.

Of course, these concerns are not exclusively linked to the healthist paradigm and the virtue-like view of healthiness (e.g. they arise from existing inequalities in resources and access to health care). However, an analysis accounting for the aristocratic view of moral life present in the virtue ethical tradition can illuminate some of the issues surrounding mHealth technologies for self-monitoring. Despite promises of greater access to health care, empowerment and cost efficiency, healthist attitudes underpinning mHealth lead to contrary effects: healthcare inequalities widen, empowerment is primarily attainable by the select few and the costs and burdens of health are disproportionately offset to the underprivileged users. As we argue in the next section, the design and implementation of mHealth need to be refashioned. The virtue-like, aristocratic understanding of healthiness is not compatible with the ideal of democratization, and we propose some design interventions, as well as conceptual changes that would help these technologies fulfill their promises.

## Healthiness and Social Justice

We argue that to reap the potential societal benefits of mHealth technologies, we must pay close attention to the similarities between healthiness as a quasi-virtue and traditional virtues not only on the level of individual agency, but also within a broader societal context. This allows us to clarify the consequences of entrenched inequalities in their relationship with the most important potential benefit of mHealth—the promotion of public health realized by the users’ combined individual health-directed endeavors. Our analysis of the relationship between healthiness as a quasi-virtue, inequalities and public health helps us to suggest some recommendations on how to overcome the issues raised in this paper.

In the Nicomachean Ethics, Aristotle emphasizes the relevance of his ethical inquiry into virtues and the objective of a virtuous life not only for the individual citizen but for the more ‘noble and godlike end’ of the goodness of the people or the city (Aristotle, 2004: 1094b). Leading a virtuous life is not an endeavor pursued by individuals for their own sake but contributes to the goodness of a wider social context. If healthiness should be conceptualized as a virtue at all, then engagement in health-related activities and the development of a healthy character should ultimately be undertaken with an outlook toward health as a social and not merely individual good. This is why campaigns encouraging individual responsibility for health by, for example, promoting individual awareness of health risks, the development of healthy habits, and active engagement in measures to maintain and promote health should be evaluated not on the basis of their impact on the health of specific persons, but the health of the society as a whole. As we argue in this paper, such an assessment cannot be made without consideration for the social justice dimension of these campaigns and the deployment of mHealth.

As we demonstrated in the previous section, the implementation and use of mHealth risk entrenching already existing inequalities. The implementation and use of mHealth assume a certain degree of digital and health literacy that is not equally distributed among those with lower education or income, which contributes to their exclusion from participation in the promotion of their own as well as the public’s health. The promise of empowerment increases the individual responsibility of the user for their own health, which requires users to expend not only financial but also temporal or emotional resources—all of which are not equally distributed within the society. Finally, the promise of cost efficiency obscures the risk of a redistribution of financial costs within the healthcare sector that benefits the wealthy while presenting a larger financial burden for those with lower resources. Independently from whether one conceptualizes social justice as equality of opportunities, sufficient capabilities, or realized outcomes for individual well-being, one can easily argue that entrenched inequalities, as we have described them, stand in contrast to social justice.

The literature on public health ethics strongly endorses the view that social justice is of primary importance for public health. This is because the health of individuals and groups is closely connected to the societal circumstances they live in. Norman Daniels argues, for instance, that we must also reduce inequalities within the social domains that have a direct or indirect impact on health in order to achieve equal opportunity and thus a socially just society (Daniels, 2008: 142–143). Scholars following the capabilities approach, like Jennifer Prah Ruger, argue that the assessment of the justice of health policies requires us to pay particular attention to their impact on health capabilities (Ruger, 2010). By this, she means the impact of health policies on the individuals’ ability to pursue valuable health goals and their ability to effectively bring them about. This does not only include measures for maintaining health directly, but also refers to social factors like education or health literacy that provide persons with the capability to act on their health. Lastly, Madison Powers and Ruth Faden argue that public health depends on the sufficient realization of different core dimensions of well-being (Powers and Faden, 2006). Being able to realize sufficiency within the core dimensions of well-being is dependent on the just arrangement of social institutions. Thus, public health cannot be separated from the project of a just society neither on the conceptual nor on the policy level.

We want to emphasize that the negative consequences of the implementation and use of mHealth for self-monitoring mean that certain groups are deprived of the opportunity to equally participate in health-related practices and are limited in their ability to achieve healthiness conceptualized as a quasi-virtue. mHealth’s lacking recognition of the social, economic, ethnic and cultural diversity of its users, as well as its potential to exacerbate existing inequalities, means that large groups of people will be unable or unwilling to conform to an elitist, aristocratic ideal of healthiness as promoted within the healthist paradigm. This lack of inclusion and social justice lies in stark contrast to the objective of public health.

## Patient Communities and the Quantified Self Movement as a Way Forward

Decision-makers must pay particular attention to the societal circumstances and social differences in which users of mHealth are embedded. We suggest an array of measures that might promote wider participation in the development and deployment of mHealth and consequently enable a larger part of the population to reap their benefits and contribute to public health.

The first obstacle to a successful implementation of mHealth for self-monitoring preventing several social groups from participating in healthiness is digital inequalities. Digital inequalities describe the differences among social groups in terms of access to digital tools as well as the ability and mode of using them (Van Dijk, 2020). By suggesting to mitigate digital inequalities, we want to emphasize the utmost importance of measures that contribute to not only digital competency, but also to sufficient internet access, and sensitivity for the different modes of use of digital and online tools across various social classes and groups. Being able to access digital tools and possessing digital literacy for using them properly are the main determinants for the successful participation in technology-driven practices. Consequently, decision-makers should recognize that even if the use of mHealth might reduce the number of hours patients spend in direct contact with healthcare practitioners, it might not be possible to guarantee the success of mHealth as a public health measure without investing time and resources into the promotion of digital literacy and the availability of technological support for the patients. Only in this way, the promise of mHealth to increase the accessibility of health services can be realized.

In addition to tackling digital inequalities, it is necessary to promote health competency among potential users of mHealth. Sufficient health competency is critical for the ability to evaluate the use of mHealth interventions, interpret health information provided by mHealth, as well as to make decisions about the necessity for further interventions, like seeking the help of a practitioner. Health competency is not only crucial for the successful use of mHealth, but it also determines whether a person engages with an app at all, since the potential benefits of mHealth can only be evaluated in the context of the knowledge about how to lead a healthy life or knowledge about specific conditions or diseases. Consequently, the deployment of mHealth technologies should be accompanied by wide educational and health-promotion initiatives that would reduce the gap between the hermeneutic capacities of various user groups. This brings the users of mHealth apps a step closer to being empowered within the medical and public health domain for it enables them to make better and more informed decisions.

Public health would also benefit from greater societal control over mHealth technologies. mHealth tools can widely differ in accuracy, purpose and accessibility which can lead to unequal outcomes and adversely impact some user groups (e.g. as more accurate devices may be typically more expensive). Moreover, consumer-grade mHealth devices do not need to meet the strict regulatory requirements applicable to medical technologies. At the very least, we suggest that states and international bodies should regulate mHealth to a much greater extent and only approve tools that guarantee adequate standards of accuracy, reliability and accessibility. At the same time, solutions such as the prescription model adopted in Germany (Sauermann *et al.,* 2022) could prove greatly beneficial from the standpoint of justice. A well-implemented prescription system for mHealth could enable public healthcare systems to set minimal requirements for mHealth technologies and make beneficial tools more affordable to a wider range of users (e.g. as the cost of a prescribed app could be covered through public healthcare funds). Applying such regulatory standards brings us closer to implementing mHealth apps in standard healthcare procedures and public health measures in compliance with other regulations such as data protection regulations. It is only by enabling this wide, systemic adoptability that the potential economic benefits of mHealth apps can be realized.

The ways in which persons are capable to interact with mHealth as well as the degree to which they are tailored to their needs are also of high relevance for the beneficial implementation of this technology. Scholars have already suggested value-sensitive design approaches to embed certain values in the technology at very early stages (Friedman *et al*., 2008; Jacobs, 2020). Such approaches provide a valuable start; however, they do not reach far enough. Value-sensitive design approaches do not necessarily include participatory initiatives that are sensitive to the modes in which different user groups might interact with the technology. Therefore, we recommend that decision-makers support the participation of marginalized user groups already at the development stage. This ensures that the diverse needs of potential users are met and increases the chances that mHealth technologies would recognize the unique circumstances in which the users function, thereby increasing the apps’ accessibility and potential to empower their users.

Even if decision-makers follow these suggestions, it is important to keep in mind that the basis for the overall successful implementation and use of mHealth in regard to their goals for public health is dependent on the wider social circumstances of their users. Health policy can only address those circumstances to a limited degree. Important factors like wealth or income, which also determine the degree to which a person can engage with mHealth, go beyond the scope of obstacles we are able to address at this point. However, increasing the sensitivity for existing inequalities and social arrangements that determine the lifeworld of potential users, might help in rendering mHealth a more successful public health measure.

Arguably, many of our suggestions aimed at institutional decision-makers can be seen as merely facilitating individual management of health without necessarily tackling the underlying social inequalities. Rather, they merely increase individuals’ health and digital literacy or reduce the cost of using mHealth *in response* to wider injustices. Admittedly, this should be seen as a limitation of our work. Despite our belief in the potential of mHealth, we must contend with it being a tool directed toward individuals and not a fully fledged public health measure. Consequently, many benefits introduced through mHealth are bound to remain at the level of the individual and it would be difficult to design interventions that would go beyond that. At the same time, a wide-reaching improvement of individual health and health literacy would scale up and provide tangible public health benefits. Consequently, mindful of the limitations of this approach, we would like to provide some thoughts on how individual self-monitoring efforts could be coordinated to better contribute to public health goals and facilitate the fulfillment of the promises of mHealth. Drawing on our view of democracy and its associated ideas of patient participation and cooperation, we discuss two recent bottom-up initiatives that we believe can play a key role in the improvement of public health through the means of mHealth.

The connectivity offered by health-related apps and devices, as well as the greater circulation of health data have led to the creation of online patient communities and the Quantified Self (QS) movement—groups that coordinate and encourage contacts between various users of mHealth and other self-monitoring technologies. Admittedly, QS has been widely criticized as contributing to the trend of individual responsibilization that we discussed in this paper (see Ruckenstein and Pantzar, 2017, for a detailed overview of the movement) and the literature discusses its impact on health perception as, at best, ambiguous (Wieczorek *et al*., 2022). However, empirical investigations into the movement and various user and patient-centric communities demonstrate that they can serve as a valuable tool for increasing public participation and interest in the provision of health care, as well as lead to feelings of solidarity and care among its members (see e.g. Barta and Neff, 2016; Sharon and Zandbergen, 2017; Kirstensen *et al*., 2021).

In our view, the discussion and sharing of health-related data among the interested parties encouraged by patient communities and QS is a valuable alternative to the individualistic and healthist attitudes promoted through mHealth. By reframing the management and monitoring of health as a shared problem that should be addressed in a participatory manner, these groups enable users to pool resources and arrive at solutions and knowledge that would not be available to isolated individuals. For example, the sharing of insights among users with different levels of health and digital literacy could help close the gap in hermeneutic capacities, while also sensitizing various involved parties to the diversity of needs, perspectives and issues associated with mHealth. While not an answer to all the issues we raised over the course of this paper, greater engagement between patients (and a possible inclusion of healthcare professionals in such bottom-up communities) could go a long way in guaranteeing a broader distribution of the benefits offered by mHealth.

Moreover, recent developments in cooperative management of health data make us hopeful about wider public health impacts of such initiatives. Data cooperatives such as MIDATA allow patients to store and steward data sourced from their mHealth tools, online interactions and medical history in shared repositories controlled through democratic procedures (Blasimme *et al*., 2018). Users who join data cooperatives enjoy collective control over aggregated datasets and are able to ensure that data is used to the benefit of users and in line with ethical and scientific standards. However, despite being primarily a data-governance solution, health data cooperatives provide several advantages that could help realize the public health benefits of mHealth.

First, as members participate in collective decision-making procedures regarding aggregated data, they need to be informed about and understand the choices involved in the stewardship of data. Consequently, active membership in a data cooperative would improve users’ digital and health literacy, especially as data cooperatives themselves can engage in activities to help users increase and utilize their hermeneutic capacities.

Second, members of data cooperatives are likely to pool their health data and contribute it for research purposes, often regardless of concerns about privacy, which could provide researchers with a greater access to a wider diversity of health data, while also giving patients more opportunities to participate in and influence medical research (Blasimme *et al*., 2018). Thanks to embeddedness in data cooperatives, isolated individual practices of self-monitoring could have an increased relevance for public health and contribute to the accomplishment of a greater range of public health goals.

Finally, membership in data cooperatives would allow patients a greater bargaining power in the context of their use of mHealth and potential reuses of their data. As organizations representing members’ interests, data cooperatives might be more successful at ensuring that patients’ needs and rights are recognized in the development and deployment of mHealth, especially since their stewardship of valuable health data would provide them with attractive bargaining chips.

## Conclusions

Healthist tendencies to treat health as an individual and individually managed problem run contrary both to the democratic promises of mHealth and to the core assumptions of the public health paradigm. To ensure that mHealth technologies for self-monitoring have a beneficial impact on public health, we should use them in ways that position health precisely as a public and not a private matter. And contrary to the virtue-like and aristocratic attitudes to health we have identified over the course of this paper, this involves more participation, more cooperation, more social justice and, ultimately, more democracy in the domain of mHealth.

For these reasons, we believe that governments and public institutions should place particular emphasis on patient participation and cooperation in the context of mHealth. By advocating for and enabling community-based initiatives such as the QS movement and patient groups or facilitating the formation and membership in data cooperatives (e.g. by mandating data portability), public bodies can overcome at least some of the limitations of the healthist paradigm within which mHealth is embedded. Due to its individualistic focus, mHealth cannot be fully understood as a public health measure, but a coordination and scaling up of individual efforts can bring tangible public health benefits.

## Footnotes

1 We refer to users generally and patients specifically, whenever the use of mHealth apps suggests a medical context that carries ethical and legal obligations.

2 It is worth noting that in this paper, we do not refer to the textbook definition of democracy as a political system in which power is held by citizens’ representatives selected in fair and equal elections. Rather, we focus on democracy as a way of organizing social life that is characterized by (equal) participation, cooperation and social justice. In this sense, the notion of democracy we discuss is more akin to the grassroots democracy endorsed by thinkers such as David Graeber (2013) or the idea of democracy as reflexive cooperation that Honneth (1998) traces back to John Dewey’s (2016) work in political philosophy. While this framing of the term may be counterintuitive, we believe that it is more in line with how democracy functions in the wider debate on mHealth. Although democratization of health and democratic promises of mHealth are not strictly defined in the literature, the ideas of democracy and democratization of health care are commonly invoked in the context of phenomena such as increased public/patient participation in the management of health (Swan, 2012; Topol, 2015; Sharon, 2017), as well as increased access and reduced health inequalities (Lucivero and Jongsma, 2017)—we discuss these in more detail in the remainder of this introductory section. Consequently, when we characterize some aspects of mHealth and healthism as undemocratic, we do not refer to their potential to undermine the rule of law or interfere with electoral processes. Rather, we focus on these developments that run contrary to the stated hopes of equal access, patient empowerment and cost efficiency.

3 Of course, higher education and wealth are also factors impacting individuals’ hermeneutic capabilities, as well as their ability to engage in the practices of healthiness and to actually achieve positive health outcomes.
